# HR-MAS NMR Applications in Plant Metabolomics

**DOI:** 10.3390/molecules26040931

**Published:** 2021-02-10

**Authors:** Dieuwertje Augustijn, Huub J. M. de Groot, A. Alia

**Affiliations:** 1 Leiden Institute of Chemistry, Leiden University, P.O. Box 9502, 2300 RA Leiden, The Netherlands; groot_h@chem.leidenuniv.nl; 2 Institute of Medical Physics and Biophysics, University of Leipzig, Härtelstr. 16–17, D-04107 Leipzig, Germany

**Keywords:** metabolomics, plants, HR-MAS NMR, multivariate analysis

## Abstract

Metabolomics is used to reduce the complexity of plants and to understand the underlying pathways of the plant phenotype. The metabolic profile of plants can be obtained by mass spectrometry or liquid-state NMR. The extraction of metabolites from the sample is necessary for both techniques to obtain the metabolic profile. This extraction step can be eliminated by making use of high-resolution magic angle spinning (HR-MAS) NMR. In this review, an HR-MAS NMR-based workflow is described in more detail, including used pulse sequences in metabolomics. The pre-processing steps of one-dimensional HR-MAS NMR spectra are presented, including spectral alignment, baseline correction, bucketing, normalisation and scaling procedures. We also highlight some of the models which can be used to perform multivariate analysis on the HR-MAS NMR spectra. Finally, applications of HR-MAS NMR in plant metabolomics are described and show that HR-MAS NMR is a powerful tool for plant metabolomics studies.

## 1. Introduction

**Figure 1:**
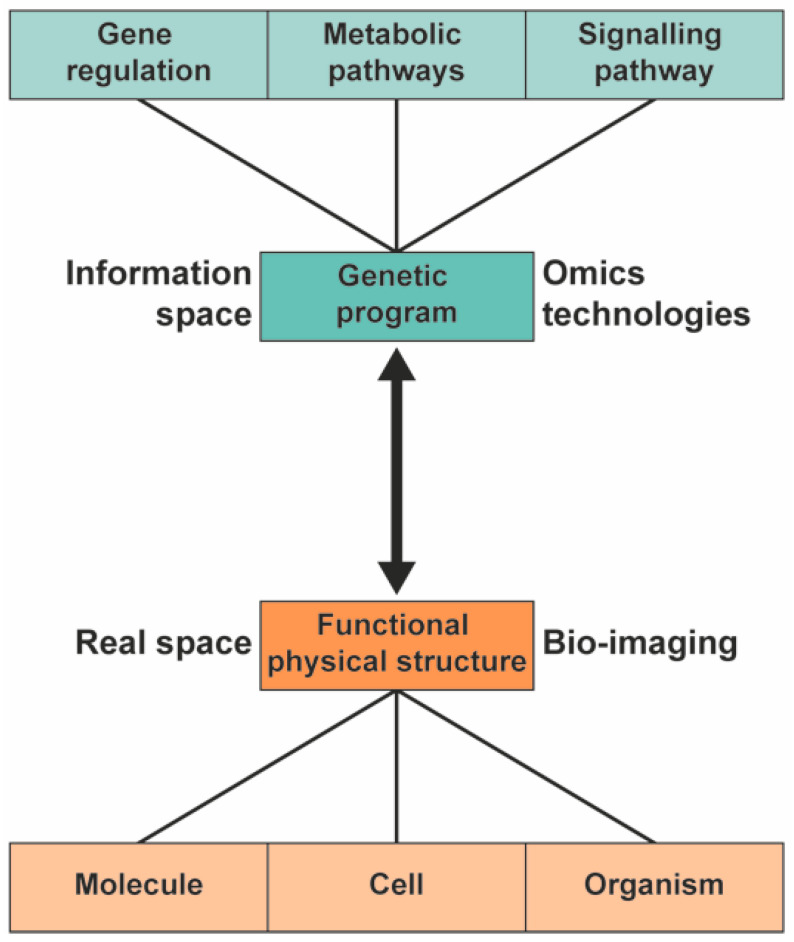
In systems biology, the information from the genetic program is integrated with information from functional physical structures to provide a comprehensive model of plants.

To understand the biological pathway underlying the phenotype of plants, a systems biology approach can be used [1,2,3]. In systems biology, the information and interaction of the functional physical structure and the genetic information are integrated to provide a comprehensive model of the organism (Figure 1). Different high-throughput technologies are used to study the genetic program of the various -omics fields: genomics, transcriptomics, proteomics, and metabolomics.

Metabolomics was the newest field added to the systems biology toolbox at the beginning of the 21st century. Metabolomics gives a quantitative and qualitative overview of all the metabolites, small molecules with a molecular weight of 30–3000 Da, present in an organism with various properties and functions [4]. There are approximately 1,000,000 different metabolites available in the plant kingdom, which makes metabolomics a challenging field [5]. Moreover, the metabolome changes quite quickly due to circadian rhythm [6,7,8] and environmental stresses [9,10] and differs between organs, tissues and even for single cells [11,12]. The metabolome is most closely related to the phenotype of a plant since metabolites are the end products of cellular processes [13]. Metabolomics is used to study development under normal and abiotic conditions (temperature, light, salt) [14] and biotic stress conditions (fungal, insects) [15,16], the safety assessment of genetically modified crops [17], speed up crop improvements [18], the effect of fruit storage [19] and the detection of food fraud [20,21].

The link between the gene regulatory network and the functional physical structure (the double arrow in Figure 1) is generally considered highly complex, with many pathways and pathway nodes interacting in what are often considered multifactorial processes. While it is undoubtedly flexible and adaptable to environmental constraints, the underlying links for a specific phenotype may turn out to be monofactorial, particularly in plants that can be grown under highly controlled conditions. The ultimate goal is to understand the complexity of organisms using metabolomics and to understand the underlying pathways of the phenotype of the organisms in a general framework [22]. This requires techniques that can study metabolomics directly in native state.

## 2. Analytical Techniques in Metabolomics

To study the metabolic profile of a plant, mass spectrometry (MS) or liquid-state nuclear magnetic resonance (NMR) spectroscopy are the most common techniques in metabolomics. Both techniques have their own advantages and limitations, as shown in Table 1. NMR spectroscopy is a method which is non-destructive, with a high reproducibility and allows to quantify metabolites. On the other hand, while MS is more sensitive, allowing to detect more metabolites in a sample, it needs different chromatography techniques such as gas chromatography (GC) or liquid chromatography (LC) for different classes of metabolites [1,2].

For both techniques, the extraction of metabolites from the sample is necessary to obtain the metabolic profile. The drawback of this extraction is that it is not only time-consuming, but also that metabolites might be lost or degraded during extraction [23]. One way to eliminate the extraction procedure is to use high-resolution magic angle spinning (HR-MAS) NMR, which allows using intact tissue samples [24,25,26].

## 3. Theoretical Background of HR-MAS NMR

An NMR experiment can be described with a nuclear spin Hamiltonian:

(1) $$ℋ\;=\;{ℋ}_{CS}+{ℋ}_{D}^{IS}+{ℋ}_{D}^{II}$$

Here:

(2) $${ℋ}_{CS}=\left\{\sigma_{iso}\gamma B_{0}+\frac{1}{2}\delta \left[3cos^{2}\left(\theta \right)-1-\eta sin^{2}\left(\theta \right)\cos\left(2\phi \right)\right]\right\}I_{z}$$

represents the chemical shift anisotropy interaction of the nuclei with the electronic environment:

(3) $${ℋ}_{D}^{IS}=\;-\frac{\mu_{0}}{4\pi }\hbar \underset{i}{\sum }\underset{j}{\sum }\frac{\gamma^{I}\gamma^{S}}{r_{ij}^{3}}\frac{1}{2}(3cos^{2}\left(\theta_{ij})-1\right)2I_{z}^{i}S_{z}^{j}\;$$

is the heteronuclear dipolar coupling between two different nuclear species *I* and *S*, and:

(4) $${ℋ}_{D}^{II}=-\frac{\mu_{0}}{4\pi }\hbar \underset{i}{\sum }\underset{j}{\sum }\frac{\gamma^{2}}{r_{ij}^{3}}\frac{1}{2}(3cos^{2}\left(\theta_{ij})-1\right)\left(3I_{z}^{i}I_{z}^{j}-I^{i}\cdot I^{j}\right)$$

is the homonuclear dipolar coupling [6,7,8].

Here, $\sigma_{iso}\;$is the isotropic value, γ the gyromagnetic ratio and η is the asymmetry parameter. For the heteronuclear and homonuclear dipolar coupling, $r_{ij}$ is the distance between the nuclei i and j, and $\theta_{ij}$ is the angle between $r_{ij}$ and the z axis. The I spin is the abundant spin and S is the rare spin. 

**Figure 2:**
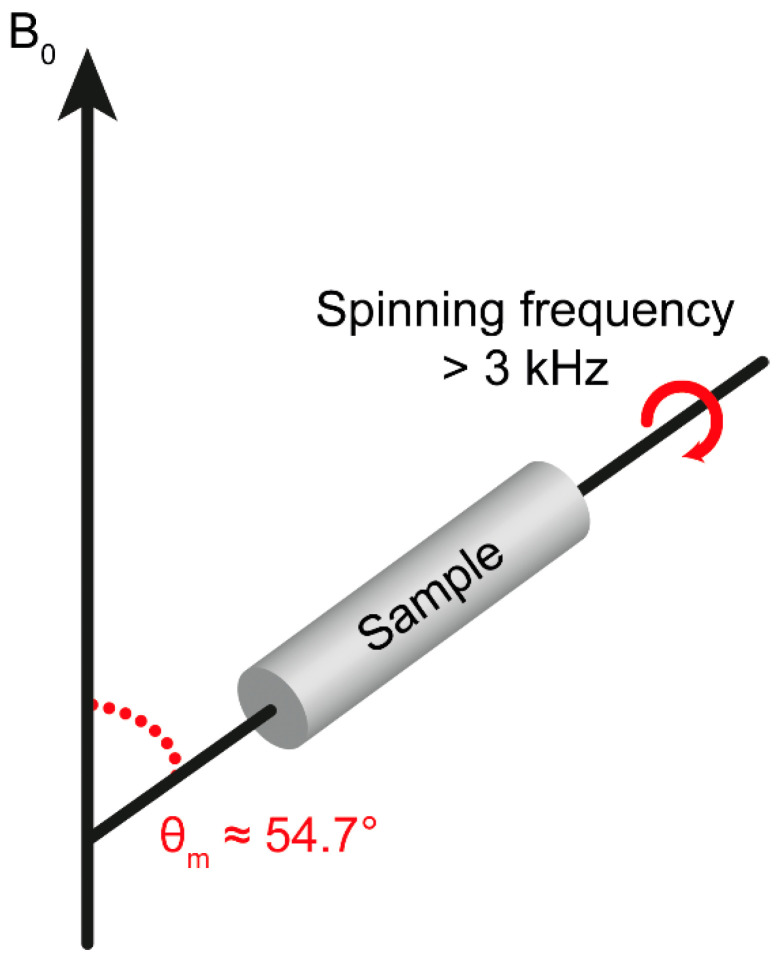
HR-MAS setup where the sample is rotated with high frequency (>3 kHz) tiled by the magic angle *θ_m_* with respect to the magnetic field (B_0_).

All three interaction terms depend on $\frac{1}{2}\left(3cos^{2}\left(\theta \right)-1\right)$, where θ is the polar angle that describes the orientation of the magnetic field $B_{0}$ in the principal axis frame of the chemical shift tensor or dipolar interaction tensor. With HR-MAS NMR, the solid sample is rapidly rotated at the magic angle $\theta_{m}=54.7{}^{\circ}$. The angular dependences of the spin Hamiltonian are averaged to zero over the sample and the broadening is effectively removed (Figure 2). Although the anisotropic interactions produce spinning sidebands, these are suppressed when spinning at high frequencies (>3 kHz), and the spectra will have narrow signals.

HR-MAS NMR is a combination of solid- and liquid-state NMR techniques, which can obtain spectra with similar resolution as spectra from liquid-state NMR experiments but make use of semi-solid samples with restricted molecular mobility [9]. Semi-solid samples, like biological tissues, can be used without extraction steps using this technique. In HR-MAS NMR, the effect of hetero- and homonuclear dipolar coupling is minimised at a frequency of a few kHz, while rigid solid samples need spinning frequencies of 20–50 kHz.

## 4. HR-MAS NMR-Based Workflow

**Figure 3:**
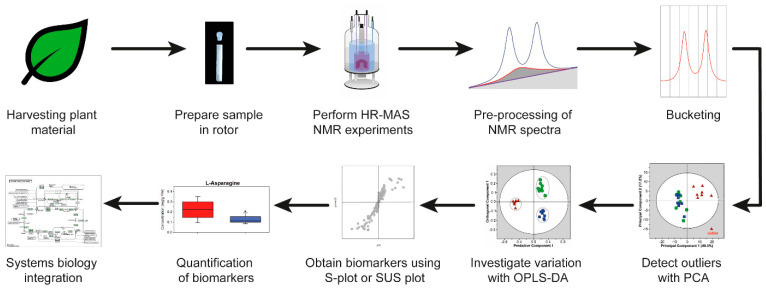
A typical high-resolution magic angle spinning (HR-MAS) NMR-based workflow. OPLS-DA, orthogonal partial least squares discriminant analysis; PCA, principal component analysis; SUS plot, Shared and unique (SUS) plot.

Here, we will explain in more detail an HR-MAS NMR-based workflow and apply the workflow to plant material. The HR-MAS NMR-based workflow is shown in Figure 3. The workflow starts with the harvesting of the leaves from plants for the preparation of a sample in the rotor, followed by performing the HR-MAS NMR experiments. The pulse sequences which can be used in metabolomics are described in Section 5. The data are pre-processed and reduced by bucketing (Section 6). Multivariate analysis is executed in three steps: the detection of outliers, investigation of the variation between different samples, and the selection of potential biomarker candidates (Section 7). Finally, the biomarkers quantification and biological interpretation is explained in a comprehensive systems biology approach by using available information from the literature. This workflow is based on a recently established liquid-state NMR approach [10]. The information about pulse sequences, the pre-processing of the data and multivariate analysis is also applicable to liquid-state NMR data. The advantage of using HR-MAS NMR spectroscopy on leaves is that experiments can be genuinely performed in vivo, which will be illustrated with selected plant metabolomics applications (Section 8). As suggested recently, sample preparations and instrumental setup protocols need to be carefully standardized in order to obtain highly reproducible and reliable data [11].

## 5. Harvesting Plant Material and Sample Preparation

For the sample preparation, it is important that the plant materials are harvested under the same controlled conditions. It is known that the light regime, time of the day, growth stage and temperature differences can affect the metabolic profile [12,13,14,15]. After harvesting, the sample should be immediately frozen in liquid nitrogen and stored at −80 °C until use [6,16,17]. For small leafy material, it is advised to directly proceed for sample packing into the zirconium rotor (as described below) before storing at −80 °C.

For the preparation of samples for HR-MAS NMR measurements, the plant material is carefully inserted into a zirconium rotor, either in intact form (for fresh samples), or by grinding the sample to powder form (for frozen samples). The space in the rotor can be minimised by using an insert. NMR reference compounds such as 3-(trimethylsilyl)-2,2’,3,3’-tetradeuteropropionic acid (TSP) or 4-4-dimethyl-4-silapentane-1-sulfonic acid (DDS) are added at this moment [11]. The rotor is then closed by putting Kel-F caps. It is important to ensure that the cap completely fits into the rotor to prevent leakages of the sample. A damaged rotor or cap should be avoided as these will interfere with stable spinning [18]. During the entire sample preparation procedure, it is important to keep the sample on ice to prevent any metabolic alternations in the sample. For different types of plant materials, it is important to standardise the sample preparation steps to prevent metabolic variation due to sample handling [6].

## 6. Pulse Sequences Used in Metabolomics

A set of pulse sequences was used in NMR-based metabolomics using both HR-MAS and liquid-state NMR spectroscopy to identify and quantify metabolites. One-dimensional spectra are mostly used to quantify metabolites. The mostly used pulse sequences are the one-dimensional ^1^H-NOESY (nuclear overhauser effect spectroscopy) with water pre-saturation and the ^1^H-CPMG (Carr–Purcell–Meiboom–Gill) sequence. NOESY spectra provide a complete and quantitative profile of the observed metabolites with the suppression of the water peak without an effect on the intensity of the other peaks [19,20,21]. CPMG is a pulse sequence which removes the broad signals from macromolecules, like proteins and lipids [19,22].

In one-dimensional NMR spectra, signals from the different metabolites strongly overlap. A way to solve this is to use two-dimensional NMR experiments. ^1^H homonuclear correlation experiments are commonly used for identification. COSY (correlation spectroscopy) identifies the spin–spin coupling of protons [19,22] and TOCSY (total correlation spectroscopy) provides information about the correlation between all protons in metabolites [20,22]. Another experiment is the ^1^H *J*-resolved where the effect of a chemical shift and *J*-coupling is separated into two independent directions [24].

With the identification of new metabolites, it is sometimes helpful to make use of ^1^H-^13^C heteronuclear correlation experiments. These experiments provide information about the coupling between a proton and a carbon [20,22]. HSQC (heteronuclear single-quantum correlation) provides input about the correlation between a proton and a carbon which are separated by one bond. In addition, HMBC (heteronuclear multiple-bond correlation) gives information about the correlation over multiple bonds [27].

## 7. Pre-Processing of One-Dimensional HR-MAS NMR Spectra

Prior to multivariate analysis and quantification, raw spectra need to be pre-processed. The pre-processing described below can be applied to spectra obtained by both HR-MAS or liquid-state NMR spectroscopy. Incorrect pre-processing can lead to spurious results [28,29]. For one-dimensional ^1^H-NMR spectrum, pre-processing involves alignment, baseline correction, bucketing, normalisation and scaling.

### 7.1. Spectral Alignment

NMR resonances can be shifted due to several factors such as changes in pH, temperature, salt concentration and inhomogeneous magnetic fields. This can give rise to variations between spectra collected from the same sample species. To solve this problem, standard chemical shifts can be used for the metabolites, and the spectra can be aligned to the standard to construct a data set for multivariate analysis [22,26]. A more elegant, unbiased protocol to align the spectra is by using an internal shift reference since this leaves the relative shifts unaffected. This is achieved by adding a reference compound with a known chemical shift with the sample. Most often, 3-(trimethylsilyl)-2,2′,3,3′-tetradeuteropropionic acid (TSP) or 4-4-dimethyl-4-silapentane-1-sulfonic acid (DDS) is used as a reference compound. Both compounds have a methyl resonance with 0 ppm chemical shift relative to tetramethylsilane (TMS), the standard reference across the entire field of ^1^H-NMR spectroscopy [28,29]. In addition, computational approaches to align the spectra have been developed in recent years [23,25]. Most of these approaches use pairwise alignment using a reference spectrum.

### 7.2. Baseline Correction

The NMR responses of metabolites are superimposed on a broad background that does not contribute any signal of interest but affects the multivariate analysis and impedes the quantification of metabolites. Polynomial-fitting of the regions in between the NMR signals is used to perform automated baseline correction [26]. After baseline correction, the spectra are truncated to have only signals from the metabolites. The region between 0.1 and 8 ppm is used for further analysis. Although water suppression is employed during acquisition, a weak remaining water signal can interfere with the multivariate data analysis and the region of the water peak around 4.8 ppm is also removed [28,29].

### 7.3. Bucketing

**Figure 4:**
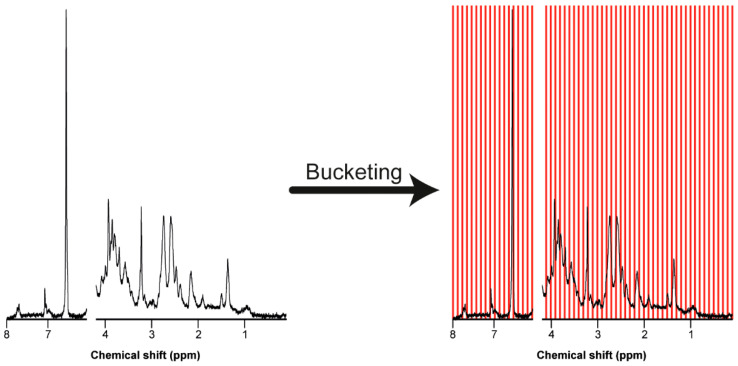
Truncated NMR spectrum before and after bucketing into equally spaced buckets of 0.04 ppm width. Bucketing allows for moderate shift averaging at the expense of resolution and provides a matrix for further processing.

The truncated NMR spectra typically have around 22.000 data points. It is common to reduce the resolution of the data by bucketing, also known as binning [26,28,29]. The most common bucketing technique is to take the area under the curve in each spaced bucket of 0.04 ppm width (Figure 4). This procedure averages minor variations in chemical shift and reduces the amount of data for the multivariate analysis [27,28,29]. However, the disadvantages of equally sized buckets or even smaller sized buckets is that a peak can split into two adjacent bins. More advanced bucketing methods, for example, adaptive-intelligent binning or adaptive binning using wavelet transforms, can be used to overcome this problem [25].

After bucketing, an i×j data matrix X is obtained with on the rows the different samples, while the columns represent the chemical shifts. The elements of the matrix contain the intensity of the bins, i.e., the signal at the different shifts for each sample.

The disadvantage of equally spaced buckets is that peaks split between two or more buckets and influence the data analysis. There are several methods, e.g., adaptive-intelligent bucketing, Gaussian bucketing, adaptive bucketing using wavelet transformation and dynamic adaptive bucking, which take into account the position of the peaks to obtain buckets with complete NMR peaks [26].

### 7.4. Normalisation 

Biological differences between preparations, for instance, different weight or dilution, result in different concentrations of specific metabolites. Normalisation methods aim to remove such systematic errors [28,29]. A standard method is to normalise the individual samples (i.e., rows) of the bucket matrix X according to:

(5) $$x_{ij}=\frac{x_{ij}}{\sum_{1}^{j}x_{i}}$$

**Figure 5:**
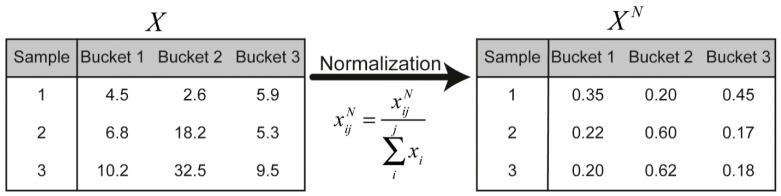
Every data point in the hypothetical bucket matrix X (i×j ) is normalised by the sum of the intensity of each sample. $x_{ij}$ is an element located in the ith row and the jth column.

This is illustrated in Figure 5 for a hypothetical case of three samples. 

Other normalisation methods include probabilistic quotient normalisation, range normalisation and normalisation to a reference metabolite [28,29].

### 7.5. Scaling 

**Figure 6:**
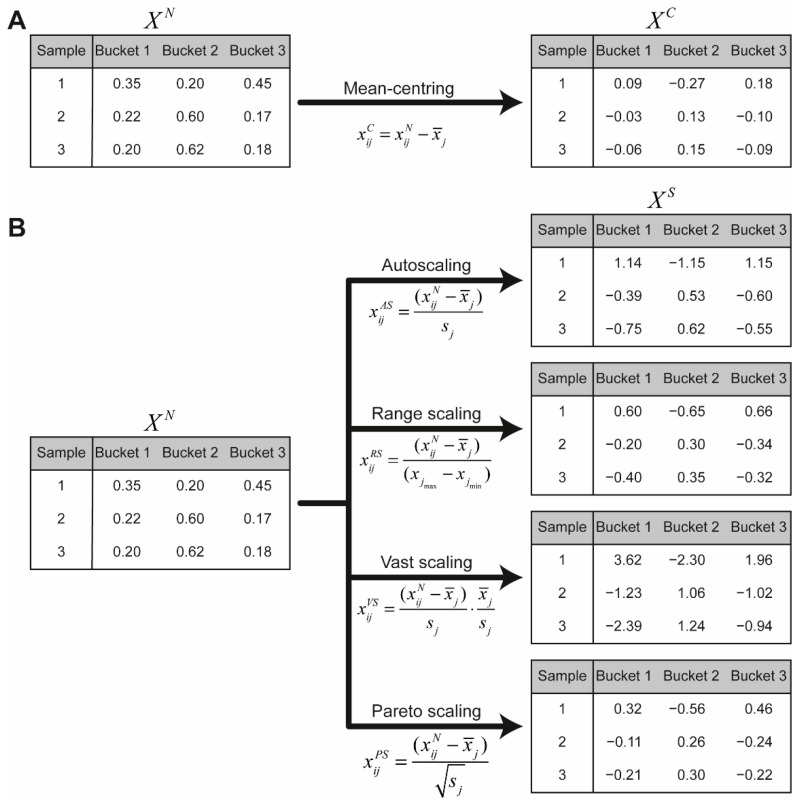
(**a**) Every column of the normalised data matrix $X^{N}$ is mean centred to obtain the data matrix $X^{C}$. (**b**) Every column in the normalised data matrix $X^{N}$ is scaled using the different methods. The obtained data matrix $X^{S}$ is used for multivariate analysis. ${\bar{x}}_{j}\;$ and $s_{j}$ are, respectively, the mean and standard deviation of the values of the jth column.

Since, metabolites present in higher concentrations contribute to the strongest variation, the scaling of the columns for selection of low abundant metabolites is necessary in the multivariate analysis [26]. The first step of scaling is the mean-centring of the samples, where the high-concentration and low-concentration metabolites are converted to values which vary around zero by subtracting the mean values from the columns (Figure 6A) [30]:

(6) $$x_{ij}^{C}=x_{ij}^{N}-{\bar{x}}_{j}$$

Scaling methods divide every bucket by a scaling factor. Scaling methods include autoscaling, range scaling, vast scaling and Pareto scaling [30,31]. Table 2 shows the different scaling factors for each scaling method and Figure 6 illustrates the different methods for the hypostatical example. More details about the scaling methods can be found in van den Berg et al. [30].

## 8. Multivariate Analysis

**Figure 7:**
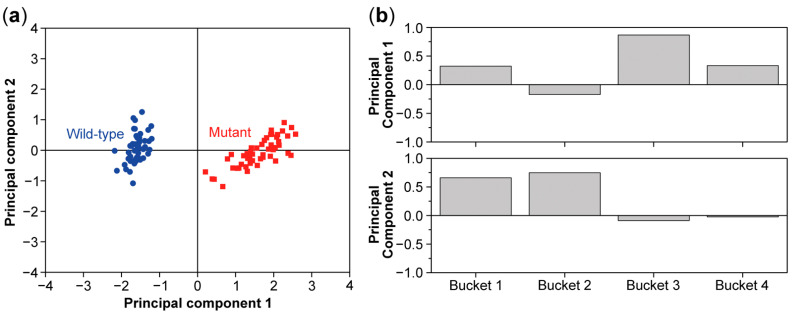
PCA score (**a**) and loading plot (**b**) of a data set including 50 wild-type samples and 50 mutant samples and 4 buckets for every sample. The score plot shows a clear separation between the wild type and mutant. The PCA loading plot shows that bucket 3 has the most influence on the first principal component and buckets 1 and 2 have the most influence on the second principal component.

Multivariate analysis considers multiple variables simultaneously to identify patterns in the HR-MAS or liquid-state NMR data corresponding to signal patterns from metabolites [26,31,32]. These generally contain more than one proton, and their signals are therefore spread over several buckets. First, unsupervised methods, methods with no assumption of any prior knowledge, are used to explore the data, find outliers and group the data [26,31,32]. One of the most used unsupervised methods is unsupervised principal component analysis (PCA), where an orthogonal transformation is used to convert the set of correlated intensities (Bucket 1, Bucket 2, …, Bucket n) with coordinates $x_{ij}^{S}$ for the samples into a set of linearly uncorrelated intensities called principal components ($PC_{1},\;PC_{2},\;\ldots ,\;PC_{n})$. PCA operates with two mathematical constraints, the largest possible variance and orthogonality. The first principal component $PC_{1}$ has the largest possible variance under the linear transformation. The subsequent vectors $PC_{i}$ are orthogonal to the preceding components and each has the highest possible variance in their coordinates under the constraints of the prior vectors $(PC_{1},\;\ldots ,\;PC_{i-1})\;$[26,31,33]. PCA converts the correlated $X^{S}$ into an uncorrelated orthogonal basis set of vector components $\left(PC_{1},\;PC_{2},\;\ldots ,\;PC_{n}\right)$, containing the scores and the new coordinates of the samples. Scores are represented in a two-dimensional score plot where each point represents a single sample on two principal component coordinates (Figure 7A). The transformation matrix that provides the information of the data after pre-processing is named the loadings; it describes how the old bucket intensities are linearly combined to the principal components and indicates which buckets have the most influences on the principal component that are represented in a loading plot (Figure 7B) [31,34,35,36]. The next step is to use databases, like the biological magnetic resonance bank (BMRB), and the human metabolome database (HMDB), to identify the metabolites corresponding to these buckets and to perform further downstream systems biology analyses [37].

Supervised methods are used to cluster the data and to determine biomarkers by following how clusters of buckets representing a specific metabolite change between e.g., the wild type and a specific mutant. The model is applied with a priori knowledge of the sample classes. Supervised methods can, therefore, be used to mark the separation between two or more sample classes at the level of individual metabolites [26,32,33]. Partial least squares discriminant analysis (PLS-DA) and orthogonal partial least squares discriminant analysis (OPLS-DA) are the most used supervised models in plant metabolomics. PLS-DA and OPLS-DA are multiple regression methods which use the pre-processed data matrix $X^{S}$ and a newly defined vector y with the value 0 for the wild-type samples and 1 for the mutants [38]. In PLS-DA, the data matrix $X^{S}$ is split into a part correlated to y and a residual part E [26,37,39,40]:

(7) $$X^{s}=X_{p}^{S}+E\;=\;T_{p}P_{p}^{T}+E$$

In OPLS-DA, the data matrix $X^{S}$ is separated into a part correlated to y, also named the predictive component ($X_{p}^{S}$), and another part that is uncorrelated to y, also called the orthogonal component ($X_{o}^{S}$) and a residual part E [26,33,34,37,40]:

(8) $$X^{s}=X_{p}^{S}+X_{o}^{S}+E\;=\;T_{p}P_{p}^{T}+T_{o}P_{o}^{T}+E$$

**Figure 8:**
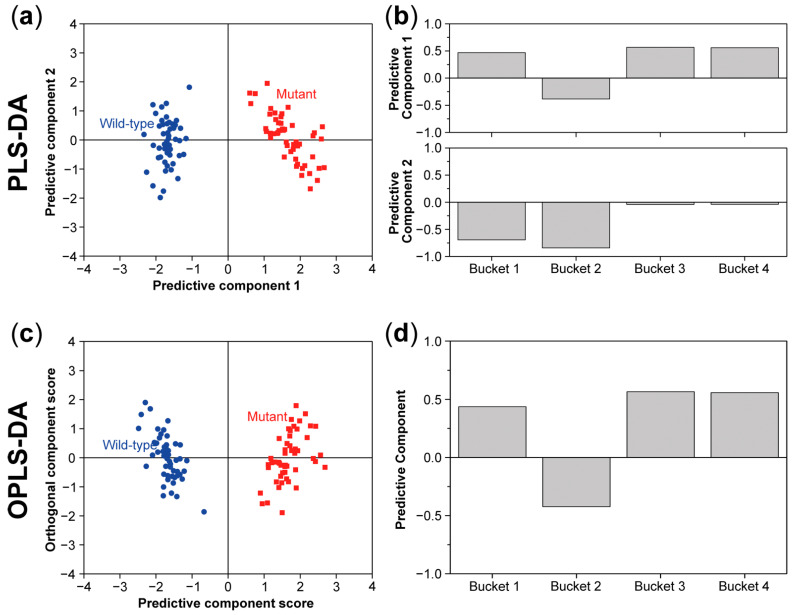
Partial least squares discriminant analysis (PLS-DA) score (**a**) and loading plot (**b**) and orthogonal partial least squares discriminant analysis (OPLS-DA) score (**c**) and loading plot (**d**) for the same data set as described in Figure 6. In both models, there is also a clear separation between the wild type and mutant in the score plots.

In both formulae above, T represents the score matrix and P the loading matrix, which can be represented, respectively, in a score plot and a loading plot (Figure 8). The loading plot of the predictive component represents the between-class variation, i.e., wild-type vs. mutants, and indicates which buckets have the strongest impact on the variation. The metabolites corresponding to these buckets are identified using metabolome databases [37].

## 9. Applications of HR-MAS NMR in Plant Metabolomics

HR-MAS NMR combined with multivariate analysis can be a powerful tool to study plant metabolomics. However, HR-MAS NMR is not used very often in plant biology. In recent decades, approximately 40 publications have reported on HR-MAS NMR-based metabolomics studies in plants. These publications are summarised in Table 3.

The HR-MAS NMR-based metabolomics studies in plants have been used for a wide range of applications. The influences of biotic and abiotic stress on the metabolic profile in plants has been widely studied by HR-MAS NMR [41,42,43,44,45,46,47,48,49,50,51,52]. Metabolomics can help in understanding developmental processes, like fruit ripening. The metabolic profile throughout the ripening process is studied in mango [53] and tomato [54]. The impact of storage time on the metabolic profile is studied on Golden Delicious apples [55] and the aging of ginseng [56]. HR-MAS NMR-based metabolomics can also be used to study the metabolic profile of specific cell types to understand the plant better. Mucci et al. studied different tissues of lemons and citrons to understand the similarities and the differences between these two fruits [57]. In addition, it is possible to use metabolic profiling to characterise newly discovered plants [58,59] or mutants of plants [39,60,61]. The geographical origin of sweet peppers [62], garlic [63] and cocoa beans [64] has been investigated with HR-MAS NMR. The original geographical origin of some food products has been certified, for example, with Protected Geographical Indications. HR-MAS NMR-based metabolomics is a useful tool for these certified products to avoid fraud [65]. Examples are the cherry tomatoes of Pachino [66,67], Interdonato lemon of Messina [67,68] and tomatoes from Almería [69]. HR-MAS NMR can also be used to determine different classes or cultivars of plants. This is helpful when only one class has a medical application as in the case of *Trichilia catigua* [70] or *Withania somnifera* [71]. It can also help to distinguish between different cultivars of apples [72], melons [73], rice [74], persimmons [75], ginseng [76], almonds [77] and curtis [78].

## 10. Conclusions and Future Perspective

High-resolution magic angle spinning NMR is a powerful tool to obtain the metabolic profile directly from plant material. The major advantage of HR-MAS NMR over liquid-state NMR is that there is no extraction step necessary which can lead to the loss of signals from non-soluble metabolites. It is a non-destructive method, which makes it possible to use the samples for other experiments such as transcriptomics analysis [79,80]. The pre-processing steps of the one-dimensional HR-MAS NMR spectra need to be done carefully. Combined with multivariate analysis, HR-MAS NMR-based metabolomics is a powerful tool to investigate plants. It is possible to link the gene regulatory network and functional physical structure, which is considered as highly complex.

It is also interesting to study the specific structures of the leaves, such as the veins, lamina or the petiole or other parts of the plants. Recently, Sarou-Kanian et al. developed a new method using ^1^H-HR-MAS slice localised spectroscopy (SLS) and HR-MAS chemical shift imaging (CSI) to determine the distribution of metabolites along the anteroposterior axis of *Drosophila melanogaster* [81]. Here, a MAS probe coupled with a three axes gradient system was used, together with pulse sequences for SLS and CSI. HR-MAS CSI is also applied to different food products and also to an intact wasp insect to examine the metabolic profile in specific regions along the sample spinning axis [82]. A slow spinning speed of 500 Hz was used to prevent damage to the insect during HR-MAS CSI measurements [83]. Due to the small sizes of specific structures of plants, high-resolution micro-MAS probe (HR-μMAS) can be considered. A lot smaller sample size (<0.5 mg) can be used in HR-μMAS in comparison to standard HR-MAS sample size (~100–150 mg) [84]. This can be used to study specific parts of plant, as shown for garlic [85].

Metabolomics provides a snapshot of the metabolic status of a sample at a specific time. For most enzymes involved in metabolism, knowledge about the in vivo kinetics is necessary to predict metabolic fluxes. Metabolic fluxes are the result of the interplay of gene expression, protein concentration, protein kinetics and regulation, and depend on metabolite concentrations. Metabolic flux analysis, also called fluxomics, can be used to determine metabolic reaction rates. Fluxomics can thus help to understand complex metabolic pathways and their regulation for the characterisation of the phenotype of the plant [86,87]. Fluxomics can be done by introducing a ^13^C-labelled precursor into the metabolic network or by supplying ^13^CO_2_ and follow the redistribution of the label into other metabolites by either NMR or mass spectrometry [88,89]. The redistribution can be followed throughout time during dynamic labelling or after reaching steady-state in a steady-state labelling approach [89]. In the current fluxomics protocols, an extraction step has to be performed, which has the disadvantage of losing components during preparation. It can thus be interesting to develop an HR-MAS NMR-based fluxomics approach which is not available at the moment.

In a multi-omics approach, the results from the various -omics technologies, such as genomics, transcriptomics, proteomics, metabolomics and fluxomics, are integrated to unravel the complexity of a biological system [90,91,92]. A major practical challenge of multi-omics is to handle different data formats and the high data dimensionality property of each data set. To integrate the different information layers, bioinformatics tools are necessary to track the different components for every layer, such as genes, proteins and metabolites at the same time [90].

## Figures and Tables

**Table 1:** The advantages and limitations of NMR spectroscopy and mass spectrometry for metabolic profiling [2,3,4,5].

	NMR Spectroscopy	Mass Spectrometry
Sensitivity	Low sensitivity, but can be improved with higher field strength and cryo- or microprobes	High sensitivity, can reach the detection limit of attomolar (10–18) concentrations
Sample measurement	In one measurement with a detectable concentration can be detected	Need chromatography techniques for different classes of metabolites
Sample recovery	Non-destructive technique Several analyses can be performed on the same extracted sample	Destructive technique
Reproducibility	Very high	Moderate
Quantification	Absolute quantitation of metabolites possible by adding one standard with known concentration	Quantification is possible with authentic standards, which are not available for newly identified compounds. Ionisation efficiencies, ion suppression and matrix effects have influences on the concentration.
Targeted or untargeted approach	Untargeted and targeted approach	Untargeted and targeted approach, mainly used for targeted analysis

**Table 2:** Overview of the scaling methods used in metabolomics [30,31]. $x_{ij}$ is an element located in the ith row and the jth column. ${\bar{x}}_{j}$ and $s_{j}$ are, respectively, the mean and the standard deviation of the values of the jth column.

Scaling Method	Formula
Autoscaling	$x_{ij}^{AS}=\frac{\left(x_{ij}^{N}-{\bar{x}}_{j}\right)}{s_{j}}$
Range scaling	$x_{ij}^{RS}=\frac{\left(x_{ij}^{N}-{\bar{x}}_{j}\right)}{\left(x_{j_{max}}-x_{j_{\min}}\right)}$
Vast scaling	$x_{ij}^{VS}=\frac{\left(x_{ij}^{N}-{\bar{x}}_{j}\right)}{s_{j}}\cdot \frac{{\bar{x}}_{j}}{s_{j}}$
Pareto scaling	$x_{ij}^{PS}=\frac{x_{ij}^{N}-{\bar{x}}_{j}}{\sqrt{s_{j}}}$

**Table 3:** Summary of the publications studying metabolomics using high-resolution magic angle spinning NMR. COSY, Correlation Spectroscopy; CPMG, Carr-Purcell-Meiboom-Gill; CPPR, composite pulses presaturation; HCA, hierarchical cluster analysis; HMBC, heteronuclear multiple bond correlation; HMQC, heteronuclear multiple-quantum correlation; HSQC, heteronuclear single quantum coherence; J-res, J-resolved, KNN, k-nearest neighbors; NOESY, nuclear Overhauser effect spectroscopy, OPLS-DA, orthogonal partial least squares discriminant analysis; PCA, principal component analysis; PLS-DA, partial least-squares discriminant analysis; STOCSY, statistical total correlation spectroscopy; TOCSY, total correlated spectroscopy.

Plant	Research Objective	Magnetic Field Strength (MHz)	Pulse Sequences	Multivariate Models
**Influences of Biotic or Abiotic Stress**				
Winter wheat (*Triticum aestivum*) [49]	Evaluate the influences of different drought treatments	400	1D	PCA
*Jatropha curcas* [50]	Determine the impacts of pruning procedures and water management	400	Zg	-
*Ribes nigrum* [51]	Determine the effect of seasonal asymmetric warming	600	CPMG, HSQC	PCA
Soybean [52]	Determine the influences of water deficiency	600	CPMG, NOESY	PLS-DA
Jatropha curcas [41]	Studying the effect of Jatropha mosaic virus on the metabolic profile	400	NOESY, CPMG, COSY	-
Pear (*Pyrus communis*) and quince (*Cydonia oblonga*) [42]	Study the effect of humic acid on the morphogenesis of pear and quince	400	^13^C, CPMG, 1D LED, COSY, TOCSY, HSQC	PCA
Lettuce (*Lactuca sativa*) [43]	Influences of the fungicide mancozeb on the leaves at different growth stages	800	NOESY, TOCSY, HSQC	PCA, PLS-DA
Tomato (*Solanum lycopersicum*) [44]	Study the influences of 6-pentyl-2H-pyran-2-one and harzianic acid on the leaves	400	CPMG, COSY, TOCSY, *J*-res, HSQC, HMBC	PCA
Maize (*Zea mays*) [45]	Determine the toxic effects on maize root tips of organo-chlorine pesticides	600	CPMG	OPLS-DA
Maize (*Zea mays*) [46]	Determine the effect of mineral or compost fertilisation and inoculation with arbuscular mycorrhizal fungi	400	CPMG, COSY, TOCSY, *J*-res, HSQC, HMBC	PCA
Soybean [47]	Determine the metabolic alternation caused by *S. sclerotiorum* infection	500	CPPR, TOCSY, HSQC	PCA
Onion (*Allium cepa L.*) [48]	Evaluate the effect of onion yellow dwarf virus on the metabolites of onions	400	Zgpr	PLS-DA
**Study the Ripening and Storage of Fruits**				
Mango fruit (*Mangifera indica*) [53]	Studying the metabolic profile of mango pulp during ripening	400	^1^H 1D, ^1^H-^13^C correlation, TOCSY, *J*-res	-
Tomato (*Solanum lycopersicum*) [54]	Studying different tissues of the tomato during fruit ripening	500	NOESY, TOCSY, HMQC	PCA
Golden delicious apples [55]	Determine the impact of storage time and production systems	500	NOESY, COSY, TOCSY	PCA, PLS-DA
Ginseng [56]	Distinguish the age of ginseng based on metabolomics	600	CPMG	PCA, PLS-DA, OPLS-DA
**Studying Different Cell Types of Plants**				
Lemon (*Citrus limon*) and citron (*Citrus medica*) [57]	The metabolic profile of different parts of the lemon and citron are studied	400	^1^H, CPMG, COSY, TOCSY, HSQC	-
**Characterising of Plant**				
*Crocus sativus* [58]	Establish the main metabolites present in *C. sativus* petals	400	^1^H, COSY, TOCSY, HSQC, HMBC	-
*Berberis laurina* (Berberidaceae) [59]	Establish the main metabolites present in *Berberis laurina* leaves, stems and roots	400	Zg, HSQC, HMBC	PCA
**Understanding Transgenic Plants**				
Poplar tree (*Populus tremula*) [39]	Studying the time- and growth-related metabolic profile of PttMYB76 and wild-type poplar tree	500	CPMG	PCA, PLS-DA
Common bean (*Phaseolus vulgaris*) [60]	Distinction between conventional and transgenic common beans	500	CPMG	PCA
“Swingle” citrumelo [61]	Evaluate the metabolic profile of non-transgenic and transgenic citrumelo	500	^1^H, HSQC, TOCSY	PCA, PLS-DA
**Geographical Origin of Plants**				
Sweet peppers (*Capsicum annum*) [62]	Discriminate sweet peppers according to their geographical origin	400	NOESY, 1D ^13^C, TOCSY	PLS-DA
Garlic (*Allium sativum*) [63]	Characterisation of two varieties garlic cropped in different Italian regions	400	NOESY, ^13^C, TOCSY, HMQC	PLS-DA
Cocoa beans [64]	Assess the geographical origins of fermented and dried cocoa beans	400	^1^H	PCA, PLS-DA, OPLS-DA
Cherry tomatoes of Pachino [66]	Determine the major metabolites present in cherry tomatoes of Pachino	700	^1^H	PCA
PGI cherry tomato of Pachino, PGI inter-donato lemon of Messina, red garlic of Nubia [67]	Identify and quantify metabolites from three typical food products of the Mediterranean diet	700	^1^H	PCA
PGI inter-donato lemon of Messina [68]	Determine metabolites unique for PGI interdonato lemon of messina	700	^1^H, COSY, TOCSY, HSQC	-
Tomato (*Lycopersicon esculentum*) [69]	Establish the metabolic differences between commercially available varieties	500	NOESY, HSQC	PCA
**Distinguish between Different Cultivars**				
*Trichilia catigua* [70]	Classification of commercial samples of Catuaba	400	CPMG	PCA, HCA
*Withania somnifera* [71]	Evaluate metabolic profile of 4 different chemotypes of *W. somnifera*	800	NOESY, CPMG, COSY, HSQC	PCA
Apples [72]	Discriminate three different apple cultivars by their metabolic profile	500	NOESY, COSY, TOCSY	PCA, PLS-DA
Melon (*Cucumis melo*) [73]	Quantification of sugars and compare two varieties	400	^1^H	-
Rice (*Oryza sativa*) [74]	Determine the metabolic variation of diverse rice cultivars	700	CPMG, TOCSY, HSQC, STOCSY	PCA, OPLS-DA
Persimmon (*Diospyros kaki*) [75]	Follow the metabolic changes during development of different cultivars	400	NOESY	PCA
Seven cultivars *of Panax ginseng* [76]	Study the primary metabolites of the seven cultivars of ginseng berries	600	CPMG	PCA, PLS-DA, OPLS-DA
Almonds (seeds of *Prunus dulcis*) [77]	Establish the difference between seven different types of almonds	500	Zg, COSY	PCA
*Curtis (Passiflora alata*) [78]	Seven herbal medicines containing leaf extract of some *Passiflora* species	500	Zg, COSY	PCA, KNN
